# miR-320a/SP1 negative reciprocal interaction contributes to cell growth and invasion in colorectal cancer

**DOI:** 10.1186/s12935-021-01874-3

**Published:** 2021-03-17

**Authors:** Wenjing Zhang, Haitao Yang, Zhongqiu Wang, Yanting Wu, Jingzhai Wang, Guihua Duan, Qiang Guo, Yu Zhang

**Affiliations:** 1 Department of Medical Oncology, The First People’s Hospital of Yunnan Province, The Affiliated Hospital of Kunming University of Science and Technology, Kunming, 650000 China; 2 Medical School, Kunming University of Science and Technology, Kunming, 650000 China; 3 Department of Radiation Oncology, Tianjin Medical University Cancer Institute and Hospital, National Clinical Research Center for Cancer, Key Laboratory of Cancer Prevention and Therapy, Tianjin’s Clinical Research Center for Cancer, Tianjin, 300060 China; 4 Department of Gastroenterology, The First People’s Hospital of Yunnan Province, The Affiliated Hospital of Kunming University of Science and Technology, Kunming, 650000 China; 5 Yunnan Provincial Clinical Medicine Center of Gastrointestinal Endoscopy, Kunming, 650000 China

**Keywords:** miRNA, Transcription factor, Interaction, MACC1, Colorectal cancer

## Abstract

**Background:**

Transcription factors (TFs) may be engaged in reciprocal regulatory circuits with certain miRNAs to maintain cellular homeostasis. Disequilibrium of the reciprocities by certain tumor-related stimuli may give rise to deregulation of downstream cellular signaling pathways, thus promoting malignant tumor phenotypes. Specificity Protein 1 (SP1) is the most representative member of the tumor-related transcription factors. Previous studies disclosed that SP1 can transcriptionally regulate miRNAs and coding genes to facilitate tumor progression. In our study, we used bioinformatic analysis to predict several SP1-binding sites within the miR-320a promoter and found that SP1 is a predicted target gene of miR-320a. Therefore, we hypothesize a reciprocal regulatory link between SP1 and miR-320a that participates in colorectal cancer (CRC) development

**Methods:**

We performed bioinformatic analysis, quantitative polymerase chain reaction (qPCR), immunoblotting, dual-luciferase reporter assays, and a series of in vitro and in vivo functional assays to describe a novel SP1/miR-320a reciprocal interaction in CRC

**Results:**

First, we found that miR-320a was significantly downregulated in CRC tissues and cell lines. Consistent with findings in other cancers, miR-320a exhibited inhibitory effects on cell growth and invasion of CRC in vitro and in vivo. Moreover, we identified SP1 as a target gene of miR-320a, and ectopic SP1 expression partly abolished miR-320a-induced inhibitory effects. Conversely, we confirmed that SP1 interacts with the miR-320a promoter, leading to depression of miR-320a. This illustrates a double-negative feedback loop between miR-320a and SP1. Additionally, based on the fact that SP1 promotes MACC1 transcription, we determined via immunoblotting that the oncogenic MACC1/MET signaling pathway was inactivated in the context of miR-320a-induced SP1 downregulation

**Conclusion:**

Taken together, our study is the first to describe a miR-320a/SP1 negative reciprocal interaction, which contributes to cell growth and invasion in CRC through modulation of the MACC1/MET signaling pathway.

## Background

Colorectal cancer (CRC) is the third most common cancer and the fourth highest in cancer-related mortality worldwide. It is estimated that global CRC burden will increase by 60% to more than 2.2 million new cases and 1.1 million deaths by 2030 [1]. Recent advances in the understanding of molecular signaling pathways have provided novel targets for anti-CRC therapy. However, it is still necessary to investigate the molecular regulatory mechanisms underlying tumorigenesis and metastasis to identify promising therapeutic targets and efficient approaches.

miRNAs represent a cluster of small non-coding RNAs acting canonically as negative regulators of gene expression via inducing mRNA degradation or inhibiting mRNA translation [2]. Emerging studies have indicated that miRNAs can promote or suppress cancerous physiological processes, depending on the target genes and downstream signaling pathways [3]. In 2008, miR-320a (previously named miR-320) was first identified to be correlated with recurrence-free survival of CRC [4]. In addition, reports have shown that miR-320a is often downregulated in an extended spectrum of cancer types including breast, liver, prostate, lung, and glioma, and is associated with patient survival and tumor stage [5–9]. Mechanistically, miR-320a may inhibit FOXM1 expression, thus suppressing FOXM1-induced epithelial-mesenchymal transition and metastasis [10, 11]. miR-320a has also been shown to negatively regulate invasion of tumor cells by blocking HMGB1 or STAT signaling [6, 12]. Silencing of miR-320a was found to be associated with Imatinib-resistance in gastrointestinal stromal tumors [13] and exacerbated chemo-resistance of breast cancer to Tamoxifen [14]. Considering the versatility of miR-320a as a cancer-related miRNA, there is a need to explore the potential mechanisms of miR-320a regulation and to identify novel miR-320a-targeting genes.

Recent studies have increasingly demonstrated the role of miR-320a in CRC progression. miR-320a is downregulated in CRC tissues compared with normal colonic epithelia [15, 16] and is associated with tumor stage and lymphatic metastasis [17, 18]. Moreover, miR-320a is correlated with sensitivity to preoperative chemoradiotherapy, while restoration of miR-320a in CRC cells can induce a shift in sensitivity [19]. Mechanistically, absence of miR-320a in CRC is responsible for enhanced cell growth, invasion, and chemo-resistance via activation of a series of tumor-promoting genes such as RAC1, SOX4, FOXM1, FOXQ1, and Wnt/β-catenin [15, 16, 20]. Notably, a genome-wide miRNA expression profiling-based analysis showed an increased expression pattern of miR-320a in colorectal adenomas with higher histologic grade [21], which indicates its regulatory effects may be different in the precancerous stage of CRC.

Dysregulation of transcription factors (TFs) in tumors induce altered gene expression and downstream signaling transduction, making them rational targets for anticancer therapy. SP1 is the most highly represented member of tumor-related transcription factors. It has been found to be overexpressed in and negatively associated with poor prognosis in a variety of tumor types [22]. Studies have shown that SP1 transactivates oncogenic genes including FAS, EGFR, VEGF, and MET, leading to enhanced activity of signaling pathways that influence cell proliferation, apoptosis, angiogenesis, and metastasis [23–26]. In addition, SP1 can interact with genes with transcriptional activity such as SMAD3 and XIAP, to form a transcription complex and mediate downstream target gene expression [26, 27]. Of note, recent reports indicate that non-coding RNA comprised of miRNA and lncRNA are also transcription targets of SP1 as well as encoding genes [28–30], suggesting a more complex SP1-centered tumor molecular regulation network than was previously thought.

Regulatory relationships between TFs and miRNAs have been reported, such as a recently identified SP1/miR-22 feedback loop in CRC that facilitates tumor progression by suppressing downstream PTEN/AKT signaling [31]. Therefore, we predicted several SP1-binding sites within the miR-320a promoter using bioinformatic analysis and found that SP1 is a predicted target gene of miR-320a. We hence hypothesized a reciprocal regulatory link between SP1 and miR-320a that participates in CRC development.

## Methods

### Bioinformatic analyses

The expression pattern of miR-320a in colon and rectal cancer tissues was analyzed using the online software OncomiR (http://www.oncomir.org/) [32], the data resource of which is obtained from The Cancer Genome Atlas (TCGA). The putative binding activity of miR-320a to the 3′UTR of SP1 mRNA was predicted using the online tools TargetScan (http://www.targetscan.org/vert_72/) and DIANA- microT-CDS (http://diana.imis.athena-innovation.gr/DianaTools/index.php?=microT_CDS/index).

### Tissue samples and cell lines

A cohort of 12 pairs of primary CRC tissues and their matched adjacent colonic mucosa were obtained from patients who underwent surgical resection at the First People’s Hospital of Yunnan Province. Tissues were snap‑frozen in liquid nitrogen followed by storage at − 80 ˚C for further use. All patients whose tissue samples were collected for the study signed an informed consent. This project was approved by the Ethics Committee of the First People’s Hospital of Yunnan Province.

Seven human CRC cell lines (HT29, SW480, SW620, LoVo, DLD-1, SW1116, and HCT116) were purchased from the Type Culture Collection of the Chinese Academy of Sciences, Shanghai, China. The human normal colonic epithelial cell line FHC was kindly donated by Dr. Liang Peng from Guangzhou Medical University (Guangzhou, China). CRC cells were cultured in RPMI-1640 medium (Invitrogen, Waltham, US) supplemented with 10% FBS (Invitrogen, Waltham, US) and 1% penicillin–streptomycin solution. FHC cells were maintained in Dulbecco’s modified Eagle’s medium (DMEM): F12 medium (Invitrogen, Waltham, US) with supplements recommended in the ATCC protocol. 293T cells were maintained in DMEM medium containing high glucose supplemented with 10% FBS. All the cells mentioned were cultured in a 37˚C humidified atmosphere containing 5% CO_2_.

### Oligonucleotide, plasmids, and cell transfection

Hsa-miR-320a mimics and the negative control oligonucleotide were synthesized by Shanghai Genepharma Co., Ltd. (Shanghai, China). For transfection, SW480 or SW620 cells were seeded into 6-well plates at a density of 3 × 10^6^ cells per well. Cells were then transfected using Lipofectamine® 2000 (Invitrogen, Waltham, US) with 30 nM miR-320a mimics or scramble controls for 48 h at 37 ˚C prior to assays or RNA/protein extraction, according to the manufacturer’s protocol.

### RNA extraction and qPCR analysis

Total RNA was isolated from CRC tissues and cells using TRIzol® reagent (Invitrogen, Waltham, US). Mature miR-320a expression in cells was determined using a Hairpin-it™ miRNAs qPCR kit (Genepharma, Shanghai, China). RNU6B was used as an endogenous control. The primer sequence for miR-320a was as follows: Forward: 5′-ATGAGAAAAAGCTGGGTTGAGA-3′, reverse: 5′-TATGGTTTTGACGACTGTGTGAT-3′. The primer sequence for RNU6B was: Forward: 5′-CAGCACATATACTAAAATTGGAACG-3′, reverse: 5′-ACGAATTTGCGTGTCATCC-3′. SP1 mRNA expression was determined using the SYBR green qPCR assay (Takara, Dalian, China). GAPDH was used as an endogenous control. The thermocycling conditions were as follows: Denaturation at 95 ˚C for 3 min followed by 40 cycles of amplification at 95 ˚C for 12 s and extension at 62 ˚C for 40 s. GAPDH was used as the endogenous control. Data were analyzed using the 2^−ΔΔCt^ method. The primer sequence for SP1 was as follows: Forward: 5′-TCATACTGTGGGAAACGCTT-3′, reverse: 5′-GACACTCAGGGCAGGCAAA-3′. The primer sequence for GAPDH was: Forward: 5′-TGACTTCAACAGCGACACCCA-3′, reverse: 5′-CACCCTGTTGCTGTAGCCAAA-3′.

### Cell function assays

Cell growth was determined using the Cell Counting Kit-8 method (MCE, Monmouth Junction, US) according to the manufacturer's protocol. For colony formation assays, cells were trypsinized and seeded in 6-well plates at a density of 3 × 10^2^ cells per well and cultured at 37˚C for 10 days. The colonies were stained with 0.1% crystal violet solution containing 80% methanol for 5 min at room temperature. Colonies with > 50 cells/colony were counted at 40× magnification using a light microscope. Cell invasion was measured using Transwell inserts with 8 μm pores (Corning, US). Cell density was adjusted to 10^6^/mL in serum-free RPMI-1640 medium and 200 μL of cell suspension was added to each upper insert pre-coated with Matrigel matrix (BD, US); 500 μL RPMI-1640 medium containing 10% FBS was added into a matched lower chamber. After a 48 h incubation, the non-invading cells were removed from the upper surface of the transwell membrane with a cotton swab and the invading cells on the lower membrane surface were fixed in methanol, stained with 0.1% crystal violet, photographed, and counted. Six random fields for each insert were observed at 100× magnification.

### Immunoblotting analysis

Cultured cells were lysed in RIPA buffer with 1% phenylmethylsulphonyl fluoride (PMSF). Protein was loaded onto an SDS-PAGE Minigel and transferred onto a PVDF membrane. After being probed with primary antibodies at 4 ˚C overnight, the blots were subsequently incubated with HRP-conjugated anti-IgG (Cell Signaling, Danvers, US). Signals were visualized using ECL substrates (Invitrogen, Waltham, US). GAPDH was used as an endogenous protein for normalization. The integrated density of all immunoblots was measured using Image J software and normalized to the density of the GAPDH internal control.

Rabbit antibodies against SP1 (#9389S), MACC1 (#86290S), c-MET (#8198S), p-AKT (#4060S), p-ERK1/2 (#4370S), ERK1/2 (#4095S), and GAPDH (#2118S) were purchased from Cell Signaling, US. A mouse antibody against AKT (#60203-2-lg) was purchased from Proteintech, US. All the primary antibodies were diluted 1:1000 for incubation.

### Lentivirus packaging and stable cell line establishment

miR-320a-expressing lentivirus particles were packaged by co-transfecting the miR-320a-expressing clone with lentiviral packaging plasmids into 293T cells using the Lenti-Pac™ HIV Expression Packaging Systems (GeneCopoeia, Rockville, US) according to the manufacturer’s instructions. For the establishment of stable miR-320a-expressing cells, SW620 cells were incubated with viral supernatant in the presence of 8 μg/mL Polybrene for 24 h, followed by puromycin (Invitrogen, Waltham, US) selection until drug-resistant colonies became visible.

### Luciferase reporter assays

To identify the complementary binding of miR-320a to the 3′UTR of SP1 mRNA, fragments of 3′UTR of SP1 containing the putative miR-320a binding site or a mutant counterpart were amplified by PCR and subcloned into a pMIR-REPORT vector immediately downstream of the luciferase gene sequence. 293T cells were plated in 24-well plates at a density of 1 × 10^5^ cells/well and then co-transfected with pMIR-REPORT constructs and miR-320a expressing vectors. Cells were harvested after 48 h incubation at 37 ˚C for further detection of luciferase activity using a dual-luciferase reporter assay system (Promega, Madison, US); results were normalized to Renilla activity.

To detect the binding activity of SP1 to the miR-320a promoter, we amplified several truncated fragments within a 2000 bp length area upstream of the miR-320a transcriptional start site (TSS) by sequential deletion. We then subcloned them into a pGL4.10 vector (Promega, Madison, US) upstream of the luciferase gene sequence. The mutant counterpart of the − 250 bp reporter was constructed by deletion mutation. To detect the binding of SP1 to the MACC1 promoter, a 500 bp length of the promoter sequence upstream of the TSS was cloned into the pGL4.10 vector (Promega, Madison, US) upstream of the luciferase gene sequence. The mutant counterpart of the reporter was constructed by deletion mutation. SW480 cells were plated in 96-well plates at a density of 5 × 10^3^ cells/well and then co-transfected with the pGL4.10 constructs and SP1 or MACC1 expressing plasmids. Cells were harvested after 48 h incubation at 37 ˚C for further detection of luciferase activity using a dual-luciferase reporter assay system (Promega, Madison, US); results were normalized to Renilla activity.

### Mouse xenograft model

Female BALB/C nude mice (4–6 weeks old) were purchased from Hunan SJA Laboratory Animal Co., Ltd (Changsha, China). The animal experiment was conducted according to protocols approved by the Committee on the Ethics of Experimental Animals of the First People’s Hospital of Yunnan Province. A total of 3 × 10^6^ cells were injected subcutaneously into the flanks of mice to generate the xenograft model (n = 4 per group). Measurement of tumor size began 10 days after cell injection when the xenografts became measurable. Tumor volume was measured every 5 days and calculated as (length [mm] × width^2^ [mm^2^])/2. The mice were sacrificed and tumors were collected at day 30. Total RNA was extracted from the xenograft tumor tissues for qPCR assays.

### Statistical analyses

Statistical analyses were performed using SPSS software version 15.0 (SPSS, Inc., Chicago, US) and GraphPad Prism software version 8.0.1 (GraphPad Software, Inc., La Jolla, US). Comparisons of miR-320a expression between CRC tissues and paired adjacent colonic tissues were performed using Wilcoxon's paired signed rank test. All data are presented as the mean ± standard deviation. Comparisons among multiple groups were performed using one-way analysis of variance followed by Tukey's post hoc test. P < 0.05 was considered statistically significant.

## Results

### miR-320a was aberrantly downregulated in CRC

**Fig. 1:**
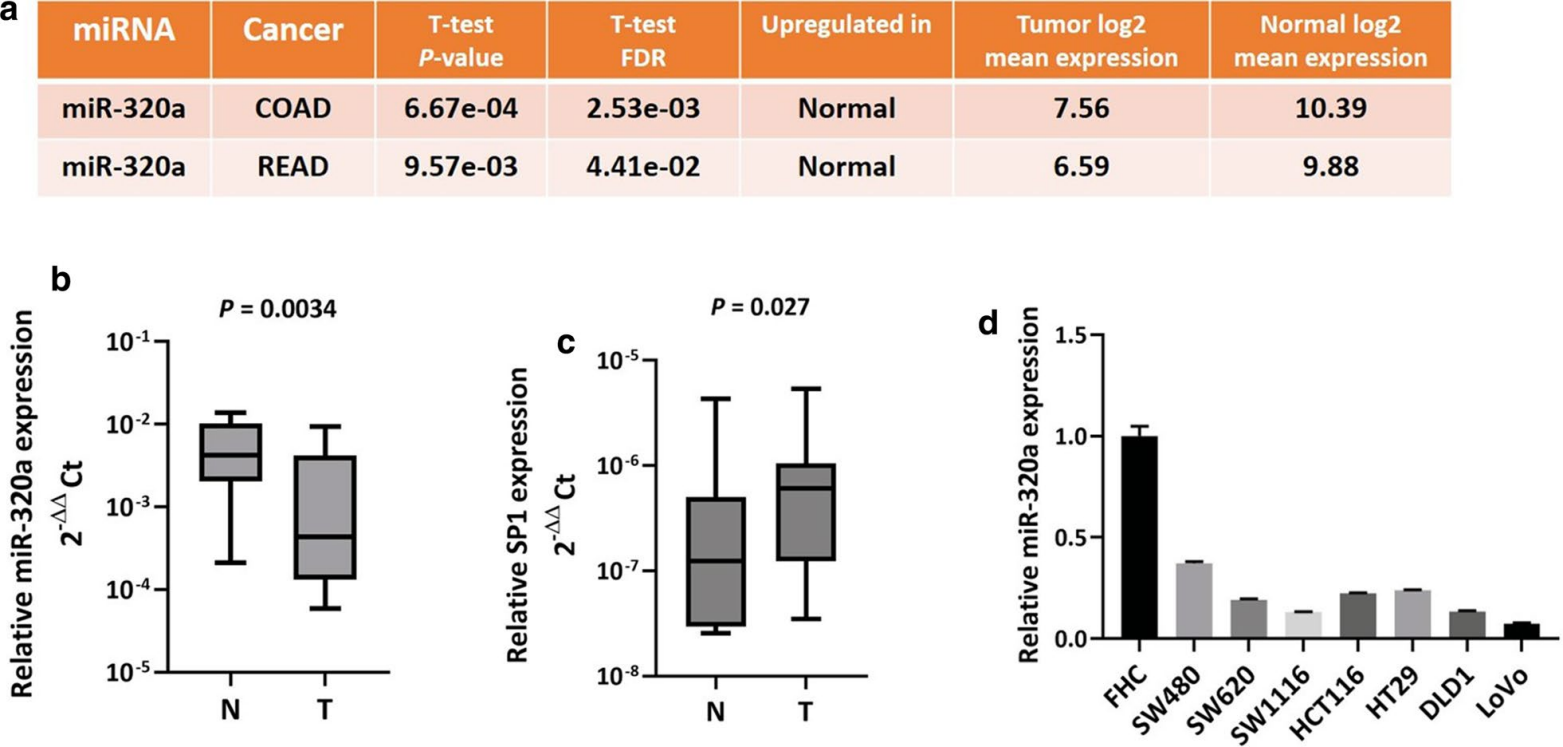
miR-320a is aberrantly downregulated in CRC tissues and cell lines. **a** In colon cancer (COAD) and rectal cancer (READ), the expression of miR-320a was significantly decreased in CRC tissues. The CRC miR-320a expression data was extracted from The Cancer Genome Atlas (TCGA) and analyzed using OncomiR online software. **b** Wilcoxon paired test showed downregulation of miR-320a (P = 0.0034) and **c** upregulation of SP1 (P = 0.027) in tissues from a cohort of 12 primary CRCs compared to their matched normal epithelial controls. **d** A commonly exhibited pattern of decreased miR-320a expression in seven CRC cell lines compared with the FHC normal human colonic epithelial cell line

miR-320a has been demonstrated to be decreased in multiple cancer types. We thus first analyzed miR-320a expression data in colorectal cancer from TCGA data using the OncomiR bioinformatic tool. As shown in Fig. 1a, miR-320a was commonly silenced in both colon cancer (COAD) (P = 6.67e−04) and rectal cancer (READ) (P = 9.57e−03) cohorts compared with matched normal adjacent epithelial tissues. We further conducted qPCR to determine miR-320a and SP1 expression in a panel of 12 pairs of clinical CRC tumors and adjacent normal epithelial specimens. Results showed that compared to adjacent normal epithelial tissues, miR-320a was significantly decreased in CRC tissues, (P = 0.0034, Fig. 1b) but SP1 was increased in CRC tissues (P = 0.027) (Fig. 1c). We also examined miR-320a expression in CRC cell lines via qPCR. The expression pattern of miR-320a across the seven tested CRC cell lines was commonly lower than in the FHC normal colonic epithelial cell line (Fig. 1d). Of the low-miR-320a expressing CRC cell lines, SW620 and SW480 were selected for further experiments.

### miR-320a inhibited CRC cell growth and invasion in vitro and tumor growth in vivo

**Fig. 2:**
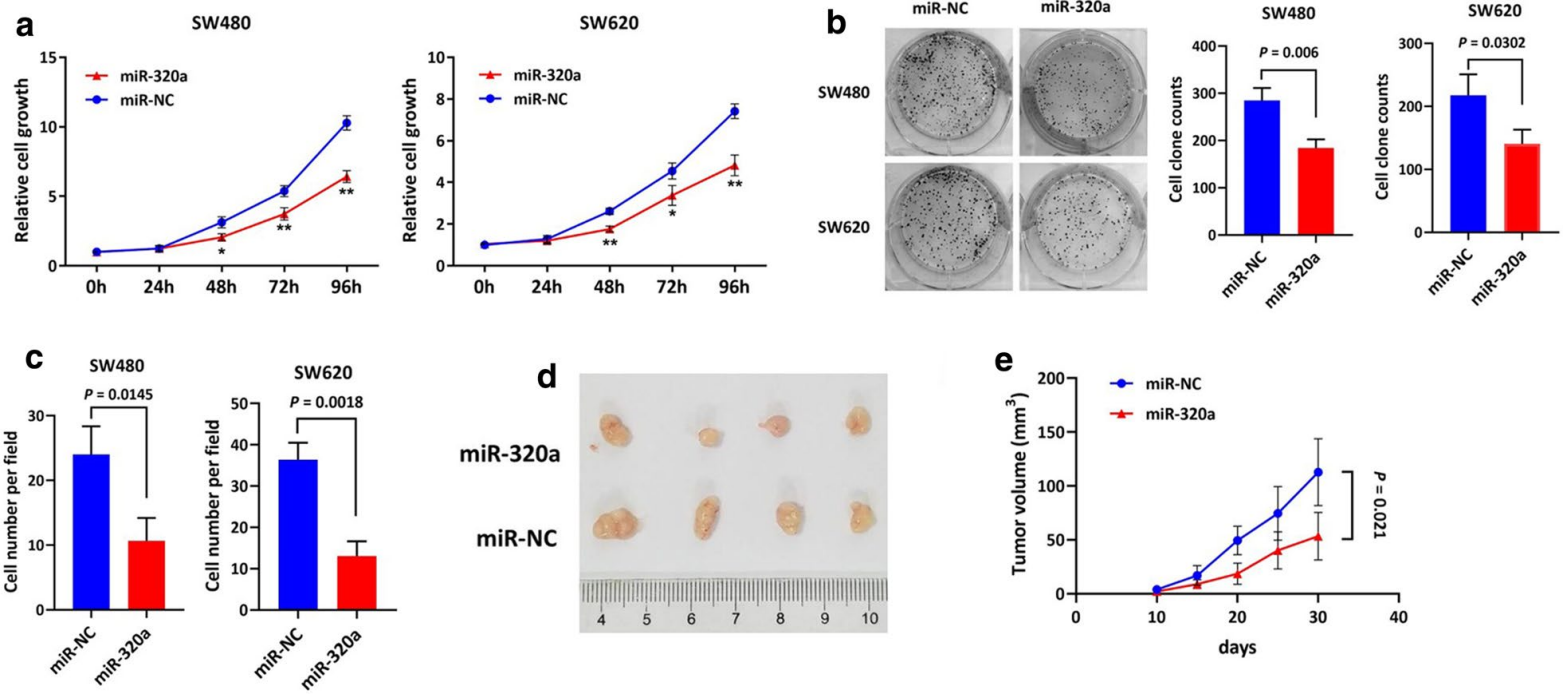
miR-320a inhibited CRC cell growth and invasion in vitro and tumor growth in vivo. **a** Restoration of miR-320a by introducing mimics significantly inhibited cell growth in SW480 and SW620 cells during a 96 h observation. *P < 0.05 vs miR-NC; **P < 0.01 vs miR-NC. **b** In colony formation assays, restoration of miR-320a caused inhibitory effects on cell colony formation in SW480 (P = 0.006) and SW620 (P = 0.0302) cells compared with negative controls. **c** Restoration of miR-320a significantly inhibited cell invasion in SW480 (P = 0.0145) and SW620 (P = 0.0018) cells. In a xenograft mouse model (n = 4 per group), mice bearing stable miR-320a-expressing SW620 cells exhibited significant reduction of tumor volume ex vivo (**d**) and tumor growth in vivo (P = 0.021) compared with negative controls (**e**)

To determine the functional relevance of miR-320a expression in modulating CRC cell malignant phenotypes, we performed in vitro gain-of-function assays by upregulating miR-320a via introducing mimics into SW480 and SW620 cells. Upon restoration of miR-320a, growth rate was significantly inhibited in both SW480 and SW620 cells compared with control counterparts (Fig. 2a). Further, restoration of miR320a induced a significant decrease in colony formation in SW480 (P = 0.006) and SW620 cells (P = 0.0302) (Fig. 2b). We also observed lower invasion of SW480 cells (P = 0.0145) and SW620 cells (P = 0.0018) as there were fewer cells adhering to the lower membrane of the transwell inserts (Fig. 2c). In a xenograft mouse model (n = 4 per group), the mice bearing stable miR-320a-expressing SW620 cells exhibited significant reduction of tumor volume ex vivo and tumor growth in vivo compared with negative controls (Fig. 2d, e). The above results indicate that miR-320a may exert suppressive effects on CRC progression.

### SP1 is a direct target gene of miR-320a in CRC

**Fig. 3:**
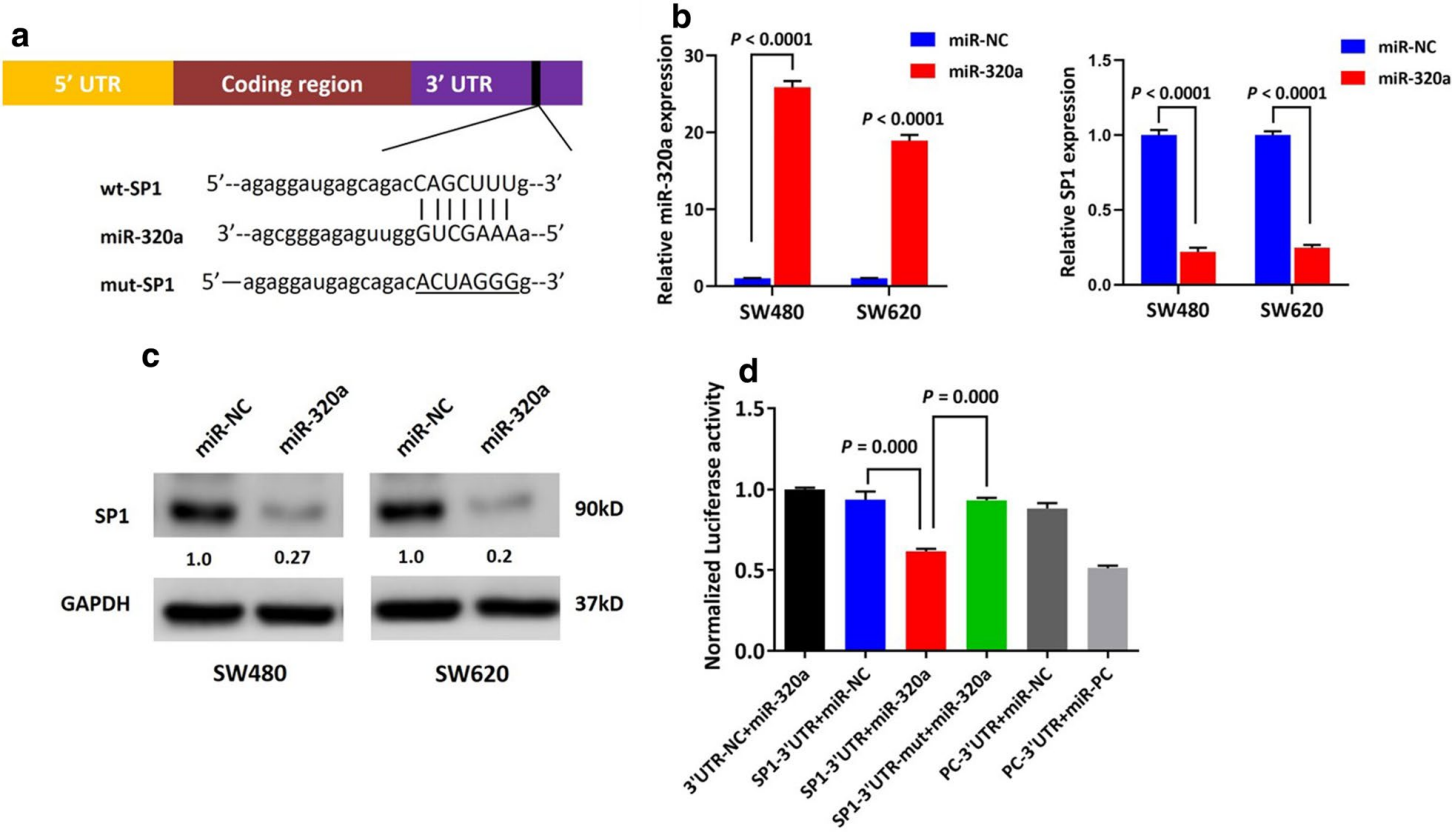
SP1 is a direct target gene of miR-320a. **a** The predicted miR-320a binding site within the SP1 3′UTR and its mutated version are presented. **b** Restoration of miR-320a in SW480 and SW620 cells induced decreased expression of SP1 mRNA as shown by qPCR assay. **c** Immunoblotting assays showed restoration of miR-320a in SW480 and SW620 cells suppressed SP1 expression at the protein level. Normalized intensity of each Western blot was analyzed and is annotated below the stripes. **d** Repression of luciferase activity by the SP1-3′UTR was dependent on miR-320a (P = 0.000), while the 3′UTR-mut of SP1 abrogated miR-320a-mediated repression of luciferase activity (P = 0.000) in 293T cells. miR-146b and TRAF6-3′UTR were used as a positive miRNA and a positive target gene 3′UTR, respectively. PC: positive control; UTR: untranslated region; Mut: mutant

It is well-known that miRNAs modulate cellular processes in tumors through negatively regulating target genes. We sought to identify the downstream target genes regulated by miR-320a. Utilizing online algorithms (TargetScan/DIANA-microT-CDS), we performed bioinformatic prediction of potential target genes of miR-320a and identified a predicted binding site for miR-320a within the 3′UTR of SP1 mRNA (Fig. 3a). We thus speculated that miR-320a may negatively regulate SP1 expression in CRC. To characterize this further, miR-320a mimics were introduced into SW480 and SW620 cells. As predicted, SP1 was decreased significantly at both the mRNA (Fig. 3b) and protein level (Fig. 3c) upon upregulation of miR-320a. To determine if the predicted miR-320a binding site within the 3′UTR of SP1 mRNA was responsible for decreased SP1 expression, we constructed a luciferase plasmid containing the wild-type 3′UTR region of SP1 (SP1-3′UTR) and a counterpart containing a mutant miR-320a binding site (SP1-3′UTR-mut). SP1-3′UTR was co-transfected with the miR-320a mimic or a negative control oligonucleotide (NC) into 293T cells and luciferase reporter assays were carried out. Results showed that miR-320a decreased luciferase activity compared to the negative control oligonucleotide (P = 0.000, Fig. 3d), while the decreased luciferase activity mediated by miR-320a was abolished upon introduction of the mutant binding site (P = 0.000, Fig. 3d). This indicates that miR-320a directly targeted SP1 by binding to the 3′UTR of SP1 mRNA.

### miR-320a inhibited CRC cell growth, colony formation, and invasion by directly targeting SP1

**Fig. 4:**
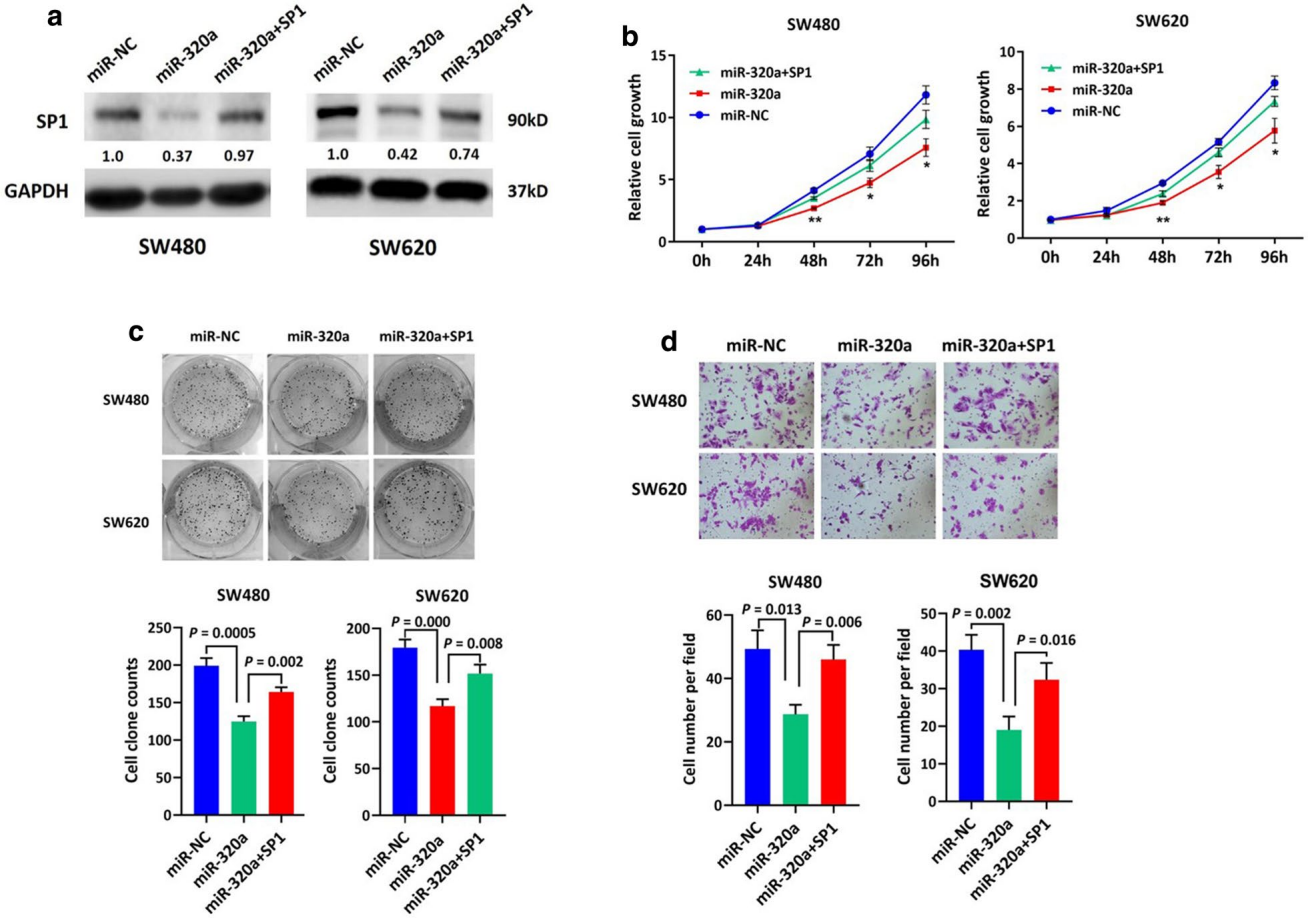
miR-320a inhibited CRC cell growth, colony formation, and invasion by directly targeting SP1. A rescue experiment was performed to determine if miR-320a exhibits tumor suppressive features by targeting SP1. **a** Immunoblotting assays showed that downregulation of SP1 expression induced by ectopic miR-320a was abrogated by transfection of an SP1-expression vector into SW480 and SW620 cells. Normalized intensity of each immunoblot was analyzed and is annotated below the stripes. Consequently, restoration of SP1 partly reversed the inhibitory influences of miR-320a on cell growth (**b**) (*P < 0.05; **P < 0.01), colony formation (**c**) and cell invasion (**d**)

Because SP1 can transactivate oncogenic genes and signaling pathways, we anticipated that miR-320a-induced downregulation of SP1 may be responsible for the suppressive effects of miR-320a restoration in CRC cells. Therefore, we performed a function rescue experiment. An SP1-expressing vector was introduced into SW480 and SW620 cells to restore SP1 expression in the presence of ectopic miR-320a expression (Fig. 4a). As expected, CCK-8 assay showed that restoration of SP1 partially relieved miR-320a-induced cell growth inhibition in SW480 and SW620 cells over 96 h of observation (Fig. 4b). Similarly, SP1 reversed miR-320a-induced inhibition of colony formation and invasion of CRC cells to a certain extent (Fig. 4c, d). These data show that miR-320a inhibited cell growth, colony formation, and invasion in CRC cells by directly targeting SP1.

### SP1 suppressed miR-320a expression transcriptionally to promote CRC cell growth, colony formation, and invasion

**Fig. 5:**
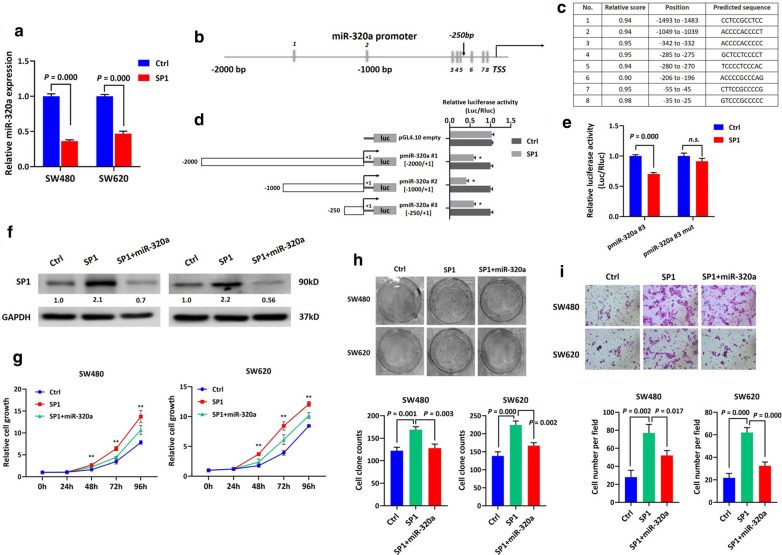
SP1 transcriptionally suppressed miR-320a expression to promote CRC progression in vitro. **a** qPCR experiments showed that miR-320a is downregulated following SP1 upregulation induced by the SP1-expression vector. **b** A diagram showing the predicted SP1 binding sites within the miR-320a promoter. **c** Sequence information of the eight putative SP1 binding sites is shown. **d** Luciferase reporter assay was performed in SW480 cells using different versions of the miR-320a promoter created by sequential deletion, as indicated by the schematic drawing. Numbers indicate nucleotide positions with respect to + 1 as the transcriptional start site. The region 250 bp upstream of the TSS of miR-320a was responsible for the SP1-incuded decrease in luciferase activity *P = 0.000; pmiR-320a: wild-type promoter sequence of miR-320a. **e** The 250 bp upstream of the miR-320a promoter predictively contained three SP1 binding sites. A mutant version of the luciferase reporter created by deletion mutation of the three binding sites was generated along with its wild-type counterpart. SP1 reduced luciferase activity by recognizing the wild-type 250 bp of the miR-320a promoter (P = 0.000); this inhibition was abrogated by the mutated counterpart. *n.s.*, not significant. Another rescue experiment was conducted by introducing miR-320a mimics into SP1-expressing SW480 and SW620 cells. **f** Immunoblotting assays showed that ectopic miR-320a expression successfully suppressed SP1 expression in the presence of the SP1-expressing vector. Normalized intensity of each immunoblot was analyzed and is annotated below the stripes. Consequently, SP1 promoted cell growth (**g**) (*P < 0.05; **P < 0.01), colony formation (**h**) and invasion (**i**) in SW480 and SW620 cells, which was partly abrogated by ectopic miR-320a expression

Previous studies reported that as an TF, SP1 regulates tumor-related miRNAs expression during tumorigenesis, which is a crucial aspect of SP1-dominated tumor modulatory mechanisms. In the current study, SP1 upregulation in SW480 and SW620 cells via an SP1-expression vector resulted in downregulation of miR-320a, implying that SP1 may affect miR-320a at a transcriptional level (Fig. 5a). The SP1 protein recognizes and binds to a consensus GC box 5′-(G/T) GGGCGG (G/A) (G/A) (C/T)-3′, termed the SP1-binding site, within the promoter region of target genes. Using the JASPAR online tool, we identified eight putative SP1 binding sites within 2000 bp of the miR-320a promoter region upstream of the TSS (Fig. 5b). The sequence information of the eight putative binding sites is shown in Fig. 5c.

To locate the enhancer element, we generated luciferase reporters containing several truncated versions of the miR-320a gene promoter sequence by sequential deletion. These constructs carry the miR-320a promoter sequence from − 2000 to + 1 (#1), − 1000 to + 1 (#2), and − 250 to + 1 (#3). Luciferase assays in SW480 cells showed that SP1 significantly decreased luciferase activity when it recognized the 2000 bp length of the miR-320a promoter sequence. Assays of the other two constructs using identical treatment conditions revealed that the region 250 bp upstream of the miR-320a TSS was responsible for the observed effect (Fig. 5d). Three putative SP1 binding sites were predicted within this 250 bp region of the miR-320a promoter (Fig. 5b). We thus generated a mutant version of the luciferase reporter containing the 250 bp promoter sequence with three invalid binding sites using deletion mutagenesis, along with a wild- type counterpart. It was observed that SP1 reduced luciferase activity when recognizing the wild-type 250 bp of the miR-320a promoter, and this inhibition was abrogated by the mutated counterpart (Fig. 5e). These findings suggest that the SP1 protein interacted with the promoter and depressed transcription of miR-320a.

To further investigate if SP1 modulates cellular processes of CRC via regulating miR-320a expression, we performed another rescue assay by introducing miR-320a into SW480 and SW620 cells in the context of SP1 upregulation. Immunoblotting showed that the upregulation of SP1 in both CRC cell types was decreased following miR-320a introduction (Fig. 5f). Consequently, function assays showed that upregulation of SP1 significantly promoted cell growth, colony formation, and invasion in the CRC cells, and this was reversed partially by miR-320a restoration (Fig. 5g–i). Our results collectively describe a reciprocal loop formed by miR-320a and SP1 which plays a regulatory role in CRC.

### miR-320a depressed oncogenic MACC1/MET signaling pathway through SP1 in CRC

**Fig. 6:**
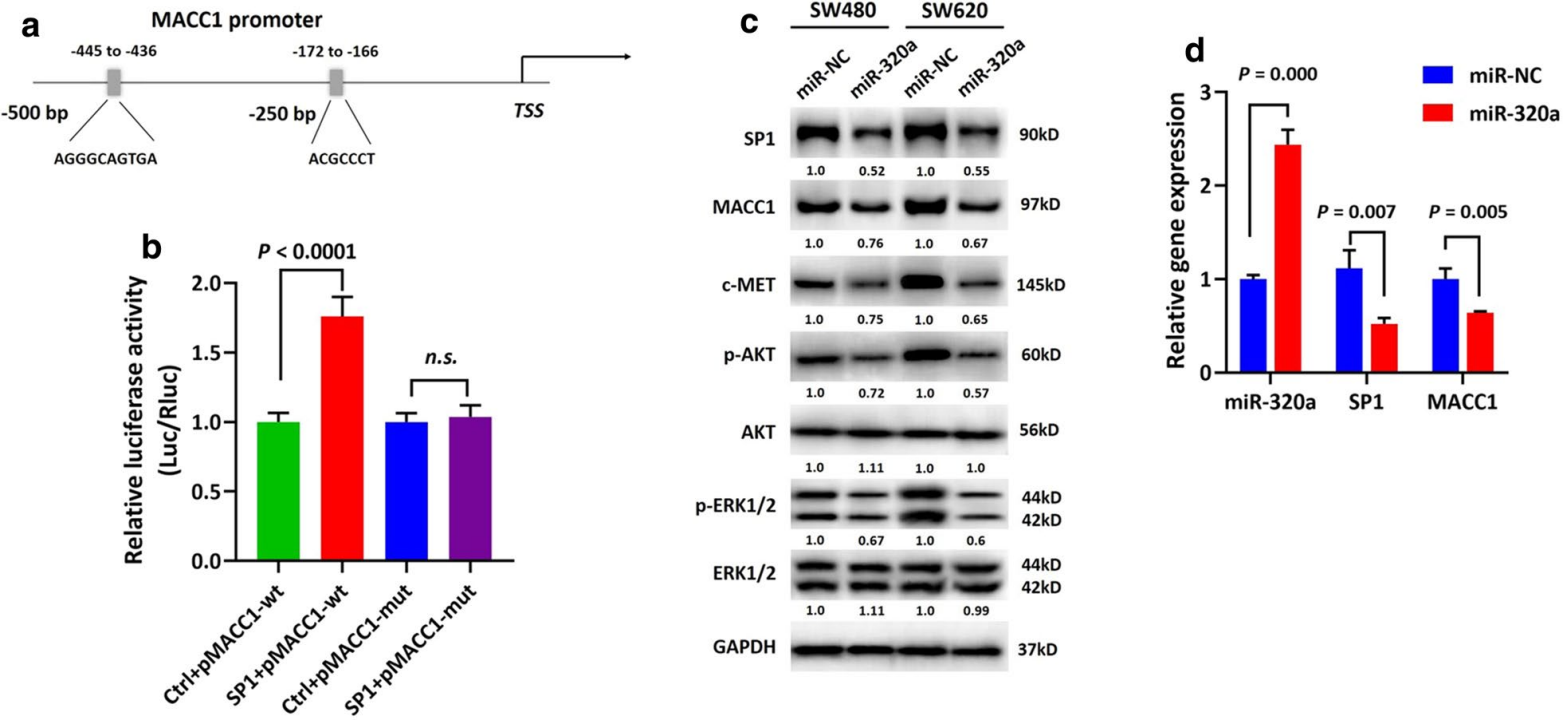
miR-320a modulated the oncogenic MACC1/MET signaling pathway through SP1. Previous study identified SP1-binding elements within the MACC1 promoter region. We performed a luciferase reporter assay to confirm whether SP1 modulates MACC1 transcriptionally. **a** A schematic diagram showing the previously identified SP1 binding site (− 172 to − 166) and a predicted one (− 445 to − 436) within 500 bp upstream of the MACC1 TSS. (B) SP1 enhanced luciferase activity by recognizing the wild-type MACC1 promoter, and the enhancement was abrogated by its mutated counterpart. (P < 0.0001). **c** Immunoblotting assays showed that ectopic miR-320a expression apparently inhibited SP1 expression followed by MACC1 inhibition. As downstream effectors of MACC1, c-MET, p-ERK1/2, and p-AKT were repressed along with ectopic miR-320a expression. Normalized intensity of each immunoblot was analyzed and is annotated below the stripes. **d** Decreased mRNA expression of SP1 and MACC1 induced by miR-320a was also observed in xenograft mouse tumor tissues. SP1: SP1 expressing vector; Ctrl: control vector; pMACC1-wt: wild-type promoter sequence of MACC1 (− 500 to + 1 bp) subcloned into a pGL4.10 luciferase reporter vector; pMACC1-mut: mutant counterpart of MACC1 promoter sequence created by deletion mutation of the two mentioned SP1 binding elements

We sought to determine the regulatory mechanism underlying the miR-320a/SP1 feedback loop by exploring its downstream effectors and signaling pathways. Metastasis Associated in Colon Cancer-1 (MACC1) has been shown to modulate tumor cell growth and activate invasion and metastasis, mainly by activation of the HGF/MET oncogenic pathway. A previous study proposed that the MACC1 promoter contains a binding element of SP1 (− 172 to − 166) which contributes to the expression of MACC1 [33]. In addition, we predicted another potential SP1 binding site (− 445 to − 436) within 500 bp upstream of the MACC1 TSS (Fig. 6a). We thus cloned a luciferase reporter containing the 500 bp wild-type sequence along with a mutant counterpart by deletion mutation of the two mentioned SP1 binding sites. We then introduced them together with the SP1-expression vector into SW480 cells. Assay results showed that SP1 increased the luciferase activity of the wild-type promoter but not the mutant one, suggesting that SP1 may enhance transcriptional activity of the MACC1 promoter (P < 0.0001, Fig. 6b). We next examined the activity of a series of crucial MACC1 downstream effectors in the context of miR-320a-induced SP1 inhibition via immunoblotting. As shown in Fig. 6c, when miR-320a was restored, suppression of SP1 led to the depression of MACC1 and c-MET expression and subsequently decreased phosphorylation and activity of ERK1/2 and AKT. This suggested that miR-320a depresses the MACC1/MET signaling pathway by directly negatively targeting SP1. In concordance with the cell culture findings, we also observed decreased mRNA expression of SP1 and MACC1 induced by miR-320a in the xenograft mouse tumors (Fig. 6d).

## Discussion

Here we show that miR-320a was commonly downregulated in CRC tissues compared with normal colonic epithelial tissues. Restoration of miR-320a suppressed cell growth, clone formation, and invasiveness of CRC cells in vitro, and tumor growth in a xenograft mouse model. miR-320a was predicted to target SP1 by binding to its mRNA 3′UTR in silico*,* this was confirmed by luciferase reporter assay and immunoblotting. Rescue of SP1 expression in CRC cells partly abrogated miR-320a-induced inhibition of cell behaviors, indicating that miR-320a exerts a tumor-suppressive role at least partly by inhibiting SP1 activity. Additionally, results showed that SP1 induced downregulation of miR-320a by binding to the miR-320a promoter and repressing transcription. Upregulation of SP1 induced decreased miR-320a expression and concomitant promotion of cell growth, clone formation, and invasiveness in CRC cells. This was partially antagonized following miR-320a mimics treatment. Moreover, mechanistic analysis showed that MACC1, previously described as a master regulator of oncogenic HGF/MET signaling [34], is a transcription target of SP1. Immunoblotting showed that MACC1 expression is repressed when SP1 is inhibited by miR-320a. As downstream effectors of MACC1, MET expression and ERK1/2 and AKT phosphor-activity were concomitantly attenuated. Our data hence illustrate a double-negative feedback loop between SP1 and miR-320a in CRC cells, which retain miR-320a expression at a low level and an elevated level of SP1, thus leading to a malignant cell phenotype by inducing sustained oncogenic MACC1/MET signaling.

Because most coding mRNAs harbor different complementary seed sequences for miRNAs recognition, a cluster of mRNAs may be regulated by a single miRNA and vice versa. Moreover, different miRNAs could co- cooperatively target a cluster of protein coding mRNAs with relevant functions or belonging to a cellular pathway [35]. In addition, from the perspective of TF-regulated gene expression, miRNA are a type of non-coding genes and presumably transcriptionally regulated by TFs. Therefore, a substantial number of regulatory interactions between TFs and miRNAs are presumably conserved across cancer entities. Recent studies have experimentally disclosed several TF-miRNA reciprocities involved in the tumorigenesis of multiple cancer types. For example, the miR-23 ~ 24 ~ 27 cluster is upregulated as an oncogene in breast cancer and can directly target HIC. HIC can in turn repress miR-23 ~ 24 ~ 27 cluster transcription, thus indicating a double negative feedback loop that promotes tumor growth [36]. In liver cancer, EZH2 is engaged in a reciprocal negative circuit with miR-101-1, thus attenuating the tumor-suppressive role by inducing downregulation of miR-101-1 [37]. In addition, interplay that implicates more than a single TF or miRNA is often described in diverse cancerous processes, especially in EMT-regulatory mechanisms. It was reported that EMT-inducing TFs such as ZEB1/2, Snail and Slug often form reciprocal regulatory networks with certain miRNAs such as the miR-34, miR-200, miR-17-92 cluster [38]. For example, the miR-200 family is induced by p53 and was the first to be identified as EMT-inhibiting miRNAs, mainly through targeting the EMT-inducing TFs ZEB1/2 by posttranscriptional modification whereas ZEB1/2 can bind to the promoters of the miR-200 family to directly repress their expression, which means frequent loss of p53 and/or miR-200 family members in tumors shifts the equilibrium of reciprocal regulation among p53, miR-200, and ZEB1/2 toward the mesenchymal state, thereby conferring the tumor cells with disseminated features [39].

It is well characterized that SP1 and its modification (phosphorylation, acetylation, and glycosylation) are crucial for maintaining cell homeostasis and the early development of embryos by retaining the basal transcriptional machinery [40]. We therefore speculate that SP1 and miR-320a reciprocity represents a manner of sustaining conventional activity of SP1 and/or miR-320a in normal cellular processes. However, the equilibrium may be shifted in tumors by tumor-related stimuli, thus giving rise to elevated levels and activity of SP1 and low levels of miR-320a expression, eventually promoting malignancy. This role change implies that SP1 is a non-oncogene addiction (NOA) gene [40], and underscores one of the causes of miR-320a downregulation in CRC. In fact, previous studies have demonstrated that SP1 activated or repressed transcription of multiple miRNAs in cancer [31, 41]. Aside from direct binding to the promoter sequence, Sp1 is reported to repress gene expression through the recruitment of histone deacetylase or DNA methyltransferase [42]. A recent study suggested that SP1 promotes migration of ovarian cancer cells via direct transcriptional repression of miR-335 or inducing hypermethylation of the miR-335 promoter [43]. This suggests that SP1-induced miRNA expression is likely a polynary mechanism.

**Fig. 7:**
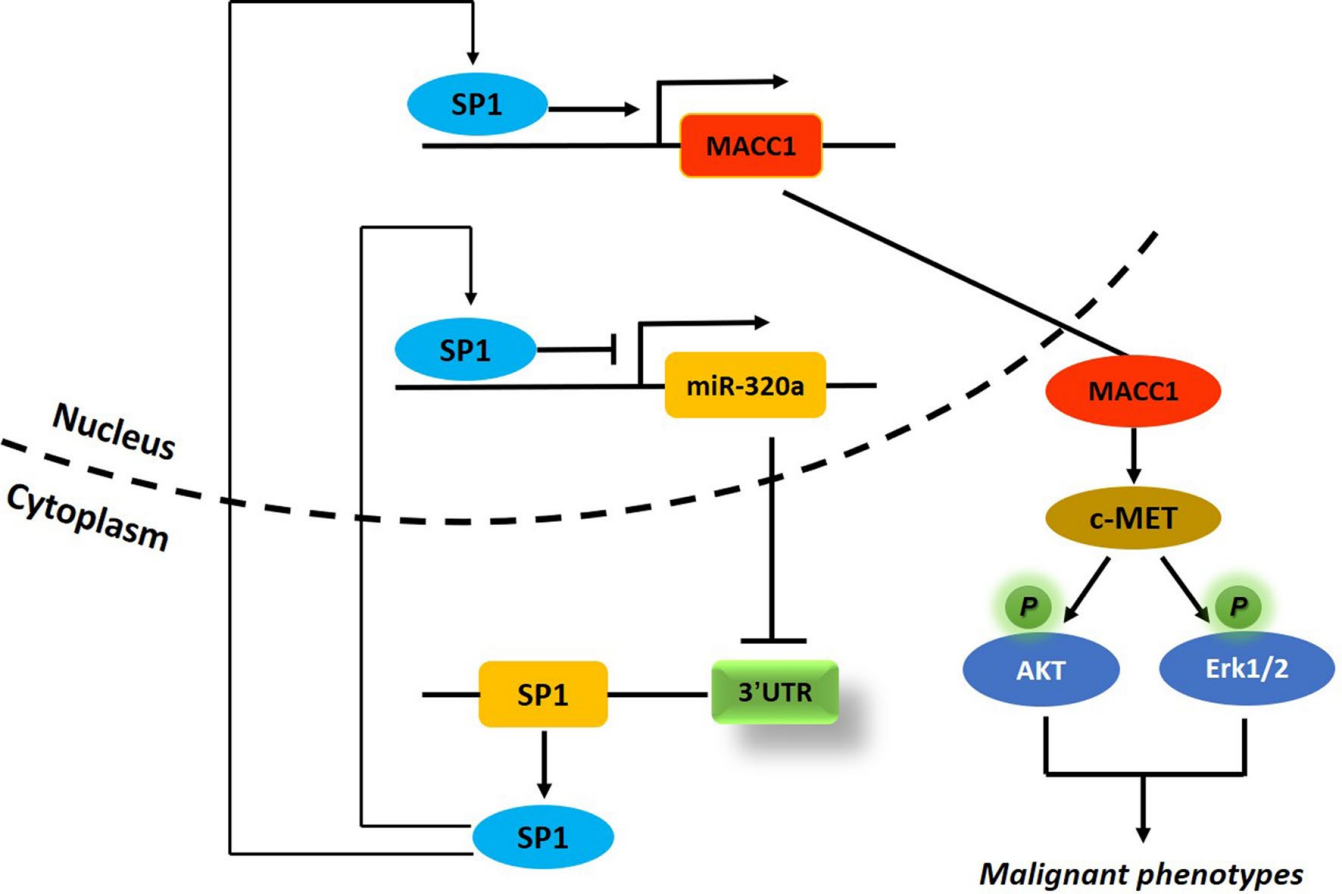
A schematic diagram of miR-320a/SP1 reciprocity engaged in modulating MACC1/MET signaling in CRC

MACC1 was established as a key player and biomarker in tumor progression first in CRC [34], then across multiple tumor entities [44]. Recent mechanistic studies have unveiled possible upstream transcriptional regulation of MACC1 by β-catenin and YB1 [45, 46], and TWIST1/VEGFA, Nanog/Oct4 axis, SPON2 [47–49] as downstream effectors, hence illustrating a MACC1-centered regulatory network in tumors. In our study, we newly describe a regulatory approach upstream of MACC1. We demonstrated that deregulation of SP1 in CRC is due to disequilibrium of SP1/miR-320a negative reciprocity, which promotes MACC1 transcription and leads to activation of MACC1-mediated c-MET and downstream signaling pathways, eventually influencing the malignant characteristics of CRC (Fig. 7). Targeting either SP1 or miR-320a may be a potential strategy to prevent MACC1-driven tumor progression.

## Conclusion

Based on these results, we propose that miR-320a forms a negative reciprocal interaction with SP1, thus potentiating cell growth and invasion of CRC through modulation of the MACC1/MET signaling pathway.

## Data Availability

All data generated or analyzed during this study are included in this published article.
